# Supplementary material for: The functional analysis of ABCG transporters in the adaptation of pigeon pea (Cajanus cajan) to abiotic stresses
Source: PeerJ. 2021 Jan 19;9:e10688. doi: 10.7717/peerj.10688 (PMC7821757; doi:10.7717/peerj.10688)
Supplement: Table S1 [file peerj-09-10688-s001.docx]

| Name | Accession | Primers-F (5’→3’) | Primers-R(5’→3’) |
| --- | --- | --- | --- |
| CcABCG5 | XP_020224844.1 | GGTGACGACGGCGTTGATGAG | CTTGTTGAAGCGTTCCTTGATGGC |
| CcABCG7 | XP_020212782.1 | GTGCATTGACCAAGCGTTCTTTCC | GCCAAGATTGAGTTGTTGCCAGTG |
| CcABCG14 | XP_020215956.1 | GACAGAGGAACCTTCTTGGCTTGG | GCCATGATTCTACCAGGCTCAGC |
| CcABCG19 | XP_020222196.1 | GGTGGGGAATGGAAAAGGGTTAGC | AATTCGCCTAGCAGTGGTTGAGTC |
| CcABCG21 | XP_020222755.1 | TGCCAGCATTGTCCCCAACTTTC | CAACCTGAAGAAGCCACCACCTAG |
| CcABCG10 | XP_020212200.1 | AATCCTGCCACATGGATGCTTGAC | ACAAGTCATTGGAACCTGGTGCTG |
| CcABCG24 | XP_020223558.1 | AGTGGCCTTGATAGTGCAGCATC | CACTGCTAGGCTGATGGATGGATG |
| CcABCG28 | XP_020225570.1 | TGCTGGAGCCTGCTTAGGATCAC | GCTGCTCATGCCAGAATCACTCTC |
| CcABCG29 | XP_020225872.1 | TGCTGAGCTACCACTCACAATTGC | ACGGTGTAAGTCCAAGGAGGATGG |
| CcABCG32 | XP_020225799.1 | CTGGATGTTGGAAGCTACCTCTGC | CTGGCGGTGGTTCACTTAACTCTC |
