# Supplementary material for: The functional analysis of ABCG transporters in the adaptation of pigeon pea (Cajanus cajan) to abiotic stresses
Source: PeerJ. 2021 Jan 19;9:e10688. doi: 10.7717/peerj.10688 (PMC7821757; doi:10.7717/peerj.10688)
Supplement: Table S2 [file peerj-09-10688-s002.docx]

| Accession | LOC | Aa | Mw | pI | Chr | Description |
| --- | --- | --- | --- | --- | --- | --- |
| KYP76113 | LOC109804364 | 1421 | 159155.33 | 7.76 | 1 | ABC transporter G family member 31-like |
| KYP75795 | LOC109806838 | 1335 | 150058.39 | 9.17 | 1 | low quality protein： ABC transporter D family member 1 |
| KYP75577 | LOC109797864 | 1520 | 170330.38 | 7.03 | 1 | putative ABC transporter C family member 15 |
| KYP75745 | LOC109818629 | 1229 | 134750.53 | 9.06 | 1 | putative multidrug resistance protein |
| KYP75435 | LOC109795230 | 1398 | 155183.72 | 6.08 | 2 | ABC transporter B family member 20-like |
| KYP74822 | LOC109795503 | 1481 | 166055.72 | 8.25 | 2 | ABC transporter C family member 10-like |
| KYP74202 | LOC109795344 | 1499 | 167987.78 | 6.72 | 2 | ABC transporter C family member 14-like |
| KYP72481 | LOC109789679 | 645 | 72010.85 | 9.08 | 2 | ABC transporter G family member 14-like |
| KYP74721 | LOC109795335 | 668 | 73512.6 | 9.47 | 2 | ABC transporter G family member 25 |
| KYP73490 | LOC109810734 | 684 | 77318.74 | 8.11 | 2 | ABC transporter G family member 26-like |
| KYP74838 | LOC109806747 | 619 | 69012.83 | 9.4 | 2 | ABC transporter G family member 4 |
| KYP75362 | LOC109815813 | 726 | 79570.36 | 8.27 | 2 | ABC transporter G family member 7 |
| KYP74261 | LOC109795045 | 333 | 36930.77 | 9.42 | 2 | ABC transporter I family member 20 |
| KYP74157 | LOC109804726 | 299 | 33528.14 | 5.56 | 2 | ABC transporter I family member 21 |
| KYP70505 | LOC109796863 | 1892 | 210837.9 | 6.51 | 3 | ABC transporter A family member 1 isoform X1 |
| KYP70901 | LOC109796091 | 1343 | 146547.84 | 7.62 | 3 | ABC transporter B family member 1 |
| KYP71725 | LOC109798062 | 1281 | 138889.96 | 7.33 | 3 | ABC transporter B family member 21-like |
| KYP71897 | LOC109796535 | 261 | 29101.29 | 6.52 | 3 | ABC transporter C family member 10-like |
| KYP69316 | LOC109796062 | 724 | 80410.82 | 6.02 | 3 | ABC transporter F family member 3 isoform X1 |
| KYP69360 | LOC109796941 | 704 | 78968.83 | 5.67 | 3 | ABC transporter F family member 5 |
| KYP70503 | LOC109797228 | 660 | 73883.51 | 8.81 | 3 | ABC transporter G family member 15-like |
| KYP71089 | LOC109796084 | 1444 | 163478.51 | 8.27 | 3 | ABC transporter G family member 34-like |
| KYP71088 | LOC109796083 | 1448 | 164560.21 | 8.28 | 3 | ABC transporter G family member 39-like isoform X1 |
| KYP71547 | LOC109796916 | 229 | 25907.34 | 9.71 | 3 | ABC transporter I family member 1 |
| KYP71090 | LOC109796805 | 1454 | 164868.63 | 7.95 | 3 | pleiotropic drug resistance protein 2-like |
| KYP68574 | LOC109798469 | 593 | 66405.12 | 6.1 | 4 | ABC transporter F family member 1 |
| KYP68041 | LOC109798752 | 718 | 80600.64 | 9.1 | 4 | ABC transporter G family member 15-like |
| KYP68044 | LOC109798893 | 686 | 76872.23 | 8.88 | 4 | ABC transporter G family member 15-like isoform X1 |
| KYP67437 | LOC109799601 | 814 | 91841.52 | 8.95 | 5 | ABC transporter G family member 18-like |
| KYP67863 | LOC109799748 | 643 | 72113.02 | 7.88 | 5 | ABC transporter G family member 5-like |
| KYP67544 | LOC109799550 | 287 | 31475.24 | 5.8 | 5 | ABC transporter I family member 6, chloroplastic |
| KYP66287 | LOC109800127 | 689 | 76475.85 | 8.79 | 6 | ABC transporter B family member 26, chloroplastic isoform X1 |
| KYP65407 | LOC109800080 | 446 | 48138.18 | 5.82 | 6 | ABC transporter B family member 28-like |
| KYP66530 | LOC109801268 | 606 | 68248.69 | 7.81 | 6 | ABC transporter E family member 2 |
| KYP66524 | LOC109801031 | 663 | 74678.78 | 8.6 | 6 | ABC transporter G family member 14 |
| KYP66379 | LOC109800436 | 721 | 81114.25 | 8.97 | 6 | ABC transporter G family member 16-like |
| KYP66999 | LOC109800523 | 1092 | 121456.99 | 8.87 | 6 | putative white-brown complex homolog protein 30 |
| KYP64698 | LOC109803084 | 1305 | 145458.66 | 5.73 | 7 | ABC transporter C family member 3 |
| KYP62452 | LOC109804060 | 1515 | 169814.5 | 8.38 | 8 | ABC transporter C family member 14-like |
| KYP62404 | LOC109804581 | 1534 | 170471.12 | 6.6 | 8 | ABC transporter C family member 5-like |
| KYP63230 | LOC109803528 | 726 | 80997.6 | 5.71 | 8 | ABC transporter F family member 4 |
| KYP61725 | LOC109804023 | 704 | 78466.57 | 8.83 | 8 | ABC transporter G family member 11 |
| KYP61369 | LOC109803314 | 1237 | 135624.84 | 9.05 | 8 | putative ABC transporter B family member 8 |
| KYP61155 | LOC109805263 | 658 | 73428.07 | 7.99 | 9 | ABC transporter G family member 11-like |
| KYP61157 | LOC109805167 | 679 | 75362.12 | 8.36 | 9 | ABC transporter G family member 11-like |
| KYP61159 | LOC109805166 | 681 | 75328.34 | 8.65 | 9 | ABC transporter G family member 11-like |
| KYP61160 | LOC109804802 | 648 | 71982.39 | 8.81 | 9 | ABC transporter G family member 11-like |
| KYP61105 | LOC109804765 | 632 | 70593.09 | 9.02 | 9 | ABC transporter G family member 9-like |
| KYP59986 | LOC109805694 | 1625 | 182829.2 | 6.92 | 10 | ABC transporter C family member 12-like |
| KYP60108 | LOC109805696 | 1104 | 123273.83 | 8.89 | 10 | ABC transporter G family member 24-like |
| KYP59743 | LOC109806282 | 156 | 18289.08 | 4.44 | 10 | ABC transporter G family member 32, partial |
| KYP58337 | LOC109805773 | 1427 | 161183.4 | 7.36 | 10 | pleiotropic drug resistance protein 1-like |
| KYP58342 | LOC109805484 | 261 | 29232.53 | 9.16 | 10 | pleiotropic drug resistance protein 1-like |
| KYP59602 | LOC109806263 | 296 | 32669.67 | 5.51 | 10 | pleiotropic drug resistance protein 1-like |
| KYP59575 | LOC109805875 | 1431 | 161968.54 | 7.97 | 10 | pleiotropic drug resistance protein 1-like isoform X1 |
| KYP55517 | LOC109808940 | 938 | 105065.15 | 8.12 | 11 | ABC transporter A family member 7-like |
| KYP57748 | LOC109808376 | 1273 | 137375.82 | 6.18 | 11 | ABC transporter B family member 11-like |
| KYP57749 | LOC109808377 | 1223 | 131323.58 | 6.32 | 11 | ABC transporter B family member 11-like |
| KYP54372 | LOC109806882 | 1496 | 164151.47 | 6.71 | 11 | ABC transporter B family member 19-like |
| KYP57750 |  | 1090 | 117234.5 | 5.59 | 11 | ABC transporter B family member 21 |
| KYP57751 | LOC109809081 | 1063 | 115211.36 | 7.23 | 11 | ABC transporter B family member 21-like |
| KYP56713 | LOC109808145 | 633 | 68549.79 | 8.79 | 11 | ABC transporter B family member 25 |
| KYP57637 | LOC109807878 | 1281 | 138151.33 | 7.59 | 11 | ABC transporter B family member 4-like |
| KYP57752 | LOC109809201 | 1226 | 132256.88 | 6.37 | 11 | ABC transporter B family member 4-like |
| KYP57754 | LOC109809415 | 1227 | 132614.51 | 6.47 | 11 | ABC transporter B family member 4-like |
| KYP56392 | LOC109807759 | 1500 | 166614.81 | 5.92 | 11 | ABC transporter C family member 3-like |
| KYP56046 | LOC109809766 | 1514 | 167661.13 | 6.39 | 11 | ABC transporter C family member 5-like |
| KYP54292 |  | 1815 | 201275.39 | 6.48 | 11 | ABC transporter C family member 8 |
| KYP54294 | LOC109807500 | 1461 | 162989.26 | 6.18 | 11 | ABC transporter C family member 8-like |
| KYP54321 | LOC109806868 | 1426 | 159138.87 | 7.87 | 11 | ABC transporter C family member 8-like |
| KYP54295 | LOC109807733 | 1464 | 163984.28 | 7.23 | 11 | ABC transporter C family member 8-like isoform X1 |
| KYP56982 | LOC109809098 | 741 | 81702.72 | 9.05 | 11 | ABC transporter G family member 22-like isoform X1 |
| KYP54262 | LOC109807460 | 1500 | 170370.83 | 6.75 | 11 | ABC transporter G family member 29 isoform X1 |
| KYP55666 | LOC109807699 | 1418 | 161820.65 | 8.9 | 11 | ABC transporter G family member 32 |
| KYP54148 | LOC109808608 | 1482 | 167729.07 | 8.19 | 11 | ABC transporter G family member 36-like |
| KYP57934 | LOC109807560 | 609 | 68159.95 | 8.65 | 11 | ABC transporter G family member 5-like |
| KYP57946 | LOC109809809 | 260 | 28339.53 | 8.24 | 11 | ABC transporter I family member 10, chloroplastic |
| KYP55322 | LOC109809826 | 1273 | 139689.35 | 8.83 | 11 | low quality protein: putative multidrug resistance protein |
| KYP53996 | LOC109808825 | 1429 | 162016.24 | 7.32 | 11 | pleiotropic drug resistance protein 1-like |
| KYP57734 | LOC109809552 | 1445 | 163218.52 | 8.03 | 11 | pleiotropic drug resistance protein 3 |
| KYP57733 | LOC109807641 | 1449 | 164070.17 | 7.67 | 11 | pleiotropic drug resistance protein 3-like isoform X1 |
| KYP36267 | LOC109790891 | 992 | 110379.05 | 8.5 | Un | ABC transporter A family member 2-like |
| KYP36268 | LOC109790896 | 944 | 105840.05 | 8.53 | Un | ABC transporter A family member 8-like |
| KYP45427 | LOC114917055 | 190 | 20607.61 | 6.41 | Un | ABC transporter B family member 11 |
| KYP33548 | LOC109792652 | 1242 | 134867.81 | 7.62 | Un | ABC transporter B family member 13-like |
| KYP34884 | LOC109791781 | 1253 | 136133.78 | 8.73 | Un | ABC transporter B family member 15-like |
| KYP41339 | LOC109819106 | 1257 | 136932.8 | 8.2 | Un | ABC transporter B family member 15-like |
| KYP41345 | LOC109819107 | 634 | 69849.44 | 8.91 | Un | ABC transporter B family member 15-like |
| KYP53100 | LOC109810515 | 722 | 79797.74 | 9.02 | Un | ABC transporter B family member 15-like |
| KYP41344 | LOC109819105 | 1143 | 126202.83 | 8.94 | Un | ABC transporter B family member 15-like isoform X1 |
| KYP33439 | LOC109792716 | 185 | 20593.32 | 9.63 | Un | ABC transporter B family member 16, partial |
| KYP50851 | LOC109812333 | 1249 | 136151.7 | 8.59 | Un | ABC transporter B family member 19 |
| KYP53791 | LOC109810109 | 1257 | 138470.31 | 8.76 | Un | ABC transporter B family member 19-like |
| KYP38572 | LOC109789325 | 1343 | 147141.35 | 7.35 | Un | ABC transporter B family member 1-like |
| KYP43375 | LOC109817706 | 748 | 81410.68 | 9.47 | Un | ABC transporter B family member 25, mitochondrial |
| KYP36469 | LOC109790742 | 366 | 40232.11 | 6.04 | Un | ABC transporter B family member 28-like |
| KYP35572 | LOC109791327 | 1243 | 136135.56 | 8.85 | Un | ABC transporter B family member 2-like |
| KYP52809 | LOC109810798 | 1241 | 136310.83 | 8.58 | Un | ABC transporter B family member 2-like |
| KYP52494 |  | 351 | 38270.17 | 6.1 | Un | ABC transporter B family member 4 |
| KYP50746 | LOC109812377 | 1402 | 155331.03 | 6.11 | Un | ABC transporter B family member 6 isoform X1 |
| KYP78557 | LOC114915696 | 246 | 26854.67 | 5.26 | Un | ABC transporter B family member 7 |
| KYP77291 |  | 335 | 36613.04 | 9.02 | Un | ABC transporter B family member 9 |
| KYP78973 | LOC109794544 | 498 | 54318.45 | 7.71 | Un | ABC transporter B family member 9, partial |
| KYP77466 | LOC109794097 | 1265 | 136849.59 | 7.8 | Un | ABC transporter B family member 9-like |
| KYP77765 | LOC109793209 | 1258 | 135844.31 | 6.57 | Un | ABC transporter B family member 9-like |
| KYP36167 | LOC114915280 | 88 | 9859.67 | 7.79 | Un | ABC transporter C family member 10 |
| KYP36168 |  | 1405 | 158768.89 | 6.87 | Un | ABC transporter C family member 10 |
| KYP49508 |  | 1212 | 136446.15 | 6.59 | Un | ABC transporter C family member 10 |
| KYP77752 |  | 238 | 27137.06 | 9.3 | Un | ABC transporter C family member 10 |
| KYP78170 | LOC109794758 | 89 | 9903.67 | 8.16 | Un | ABC transporter C family member 10 |
| KYP78893 | LOC109794812 | 256 | 29256.83 | 6.06 | Un | ABC transporter C family member 10 |
| KYP79021 |  | 382 | 42459.68 | 5.28 | Un | ABC transporter C family member 10 |
| KYP36169 | LOC109790960 | 947 | 107207.74 | 8.9 | Un | ABC transporter C family member 10-like |
| KYP77288 | LOC109794862 | 156 | 16857.33 | 6.58 | Un | ABC transporter C family member 10-like |
| KYP35744 | LOC109791194 | 1479 | 167035.52 | 7.19 | Un | ABC transporter C family member 10-like isoform X1 |
| KYP50440 | LOC109812635 | 1467 | 163999.54 | 5.96 | Un | ABC transporter C family member 13 isoform X1 |
| KYP51014 | LOC109812219 | 1514 | 169845.64 | 8.05 | Un | ABC transporter C family member 14-like isoform X1 |
| KYP46824 | LOC109815306 | 1619 | 181845.51 | 6.54 | Un | ABC transporter C family member 2-like |
| KYP42184 | LOC109818566 | 1502 | 167467.14 | 7.24 | Un | ABC transporter C family member 3-like |
| KYP35331 | LOC109791478 | 1523 | 170413.14 | 6.77 | Un | ABC transporter C family member 3-like isoform X1 |
| KYP38847 | LOC109789109 | 1536 | 171866.81 | 6.68 | Un | ABC transporter C family member 3-like isoform X1 |
| KYP38849 | LOC109789111 | 1305 | 145234.21 | 6.3 | Un | ABC transporter C family member 3-like isoform X1 |
| KYP51015 | LOC109812220 | 1496 | 168168.85 | 8.02 | Un | ABC transporter C family member 4-like |
| KYP78344 | LOC109794339 | 669 | 75459.34 | 9.11 | Un | ABC transporter C family member 8-like |
| KYP43723 | LOC109817451 | 1337 | 150197.28 | 9.05 | Un | ABC transporter D family member 1-like |
| KYP43273 | LOC109817805 | 524 | 58701.86 | 5.16 | Un | ABC transporter D family member 2, chloroplastic |
| KYP51785 | LOC109811612 | 598 | 66944.66 | 6.1 | Un | ABC transporter F family member 1-like |
| KYP51380 | LOC109811918 | 612 | 68365.89 | 9.39 | Un | ABC transporter G family member 10 |
| KYP34490 |  | 197 | 21521.72 | 6.2 | Un | ABC transporter G family member 2 |
| KYP35755 | LOC109791193 | 189 | 19665.1 | 11 | Un | ABC transporter G family member 2, partial |
| KYP48157 | LOC109814303 | 678 | 75455.58 | 9.06 | Un | ABC transporter G family member 21-like |
| KYP53034 | LOC109810595 | 681 | 75466.37 | 8.91 | Un | ABC transporter G family member 21-like |
| KYP49445 | LOC109813363 | 744 | 82372.18 | 8.91 | Un | ABC transporter G family member 22-like isoform X1 |
| KYP52347 | LOC109811227 | 651 | 73181.09 | 8.45 | Un | ABC transporter G family member 23 isoform X1 |
| KYP53068 | LOC109810569 | 1115 | 123587.99 | 9.16 | Un | ABC transporter G family member 24-like isoform X1 |
| KYP49769 | LOC109813109 | 683 | 77708.58 | 9.05 | Un | ABC transporter G family member 26-like |
| KYP37548 | LOC109790064 | 1087 | 120433.13 | 8.96 | Un | ABC transporter G family member 28-like |
| KYP51780 | LOC109811628 | 723 | 80384.61 | 8.89 | Un | ABC transporter G family member 3 |
| KYP52287 | LOC109811196 | 1229 | 138171.93 | 8.81 | Un | ABC transporter G family member 31-like |
| KYP31100 |  | 177 | 20703.59 | 9.71 | Un | ABC transporter G family member 32 |
| KYP33302 | LOC109792780 | 308 | 35071.58 | 4.69 | Un | ABC transporter G family member 32 |
| KYP33305 | LOC109792779 | 308 | 35071.58 | 4.69 | Un | ABC transporter G family member 32 |
| KYP47707 |  | 119 | 13976.41 | 6.7 | Un | ABC transporter G family member 32 |
| KYP41536 | LOC109818995 | 1445 | 163678.86 | 8.75 | Un | ABC transporter G family member 39-like isoform X1 |
| KYP47060 | LOC109815112 | 755 | 83546.16 | 9.22 | Un | ABC transporter G family member 6-like |
| KYP39219 | LOC109788852 | 624 | 69345.94 | 8.87 | Un | ABC transporter G family member 9 |
| KYP32835 | LOC109793033 | 628 | 70573.41 | 8.74 | Un | ABC transporter G family member 9-like |
| KYP40929 | LOC109787668 | 294 | 32894.99 | 9.01 | Un | ABC transporter I family member 11, chloroplastic isoform X1 |
| KYP77413 | LOC109794461 | 257 | 28110.65 | 5.36 | Un | ABC transporter I family member 17 |
| KYP40544 | LOC109787945 | 229 | 25893.32 | 9.71 | Un | ABC transporter I family member 1-like |
| KYP34407 | LOC109792126 | 585 | 66105.72 | 8.47 | Un | pleiotropic drug resistance protein 1-like |
| KYP36564 | LOC109790672 | 1457 | 164896.42 | 8.25 | Un | pleiotropic drug resistance protein 1-like isoform X1 |
| KYP35274 | LOC109791517 | 1473 | 167555.55 | 7.74 | Un | pleiotropic drug resistance protein 2-like isoform X1 |
| KYP48955 | LOC109813737 | 1358 | 154173.34 | 8.61 | Un | pleiotropic drug resistance protein 2-like isoform X1 |
| KYP52974 | LOC109810708 | 1439 | 161791.59 | 7.1 | Un | putative ABC transporter C family member 15 |

Note.

Aa，Amino acid；Mw，Molecular weight；pI，Isoelectric point；Chr，Chromosome.
