# Supplementary material for: The functional analysis of ABCG transporters in the adaptation of pigeon pea (Cajanus cajan) to abiotic stresses
Source: PeerJ. 2021 Jan 19;9:e10688. doi: 10.7717/peerj.10688 (PMC7821757; doi:10.7717/peerj.10688)
Supplement: Table S3 [file peerj-09-10688-s003.docx]

| Name | Sequences | Annotate1 | Annotate2 | Annotate3 |
| --- | --- | --- | --- | --- |
| Motif1 | GIIKPSRMTLLLGPPSSGKTTLLLALAGKLDKDLKVSGRITYNGHELNEFVPQRTSAYISQHDLHIGEMTVRETLDFSARCQGVGTR |  | P_loop_NTPase structure | ABC_transporter_like |
| Motif2 | EMJVGPAKALFMDEISTGLDSSTTFQIVKCLRQMVHIMDGTAVISLLQPAPETFELFDDIILLSEGQIVYQGPRENVLEFFESMGFKCPERKGVADFLQEVTSKKDQEQYWARKDEPYRYVSV |  | P_loop_NTPase structure |  |
| Motif3 | HPAALVKKKYGISKWELLKACFSREWLLMKRNSFVYIFKTSQJTIVAJITMTVFLRT |  |  |  |
| Motif4 | FNLFSGFFIPRPKIPIWWRWYYWISPVAWTLYGLLTSZYGD |  | P_loop_NTPase structure | ABC_transporter_like |
| Motif5 | KQJLKGVTGAFRPGEJTALMGPSGAGKTTLLDVLAGRLTGG |  | P_loop_NTPase structure | ABC_transporter_like |
| Motif6 | SGYPKKQETFARISGYVEQBDILSPHLTVEETLLFSAWLRL | ABC_2_trans structure |  |  |
| Motif7 | VRGJSGGZRKRVSIGVEJVANPSJJFLDEPTSGLDSTAAAIVMR |  |  |  |
| Motif8 | TVVCTIHQPSSRIFELFDELJLLSRGGTVYYG | ABC_2_trans structure |  |  |
| Motif9 | GPLGRHSHKLIEYFEAIPGVPKIKDGYNPATWMLEVTSPAVEARLGVDFAEIYKNSELYRRNKELIKELSTPPPGSKDLY |  |  |  |
| Motif10 | FPTKYSQSFLVQCKACLWKQHLSYWRNPPYNAVRFFFTIVIALLFGTIFWDKGKKREKZQDLFNVLGAMYAAVLFLGVNNSSSVQPVVAIERTVFYRERAAGMYSALPYAIAQ |  |  |  |
