# Supplementary figures and images for: The functional analysis of ABCG transporters in the adaptation of pigeon pea (Cajanus cajan) to abiotic stresses
Source: PeerJ. 2021 Jan 19;9:e10688. doi: 10.7717/peerj.10688 (PMC7821757; doi:10.7717/peerj.10688)

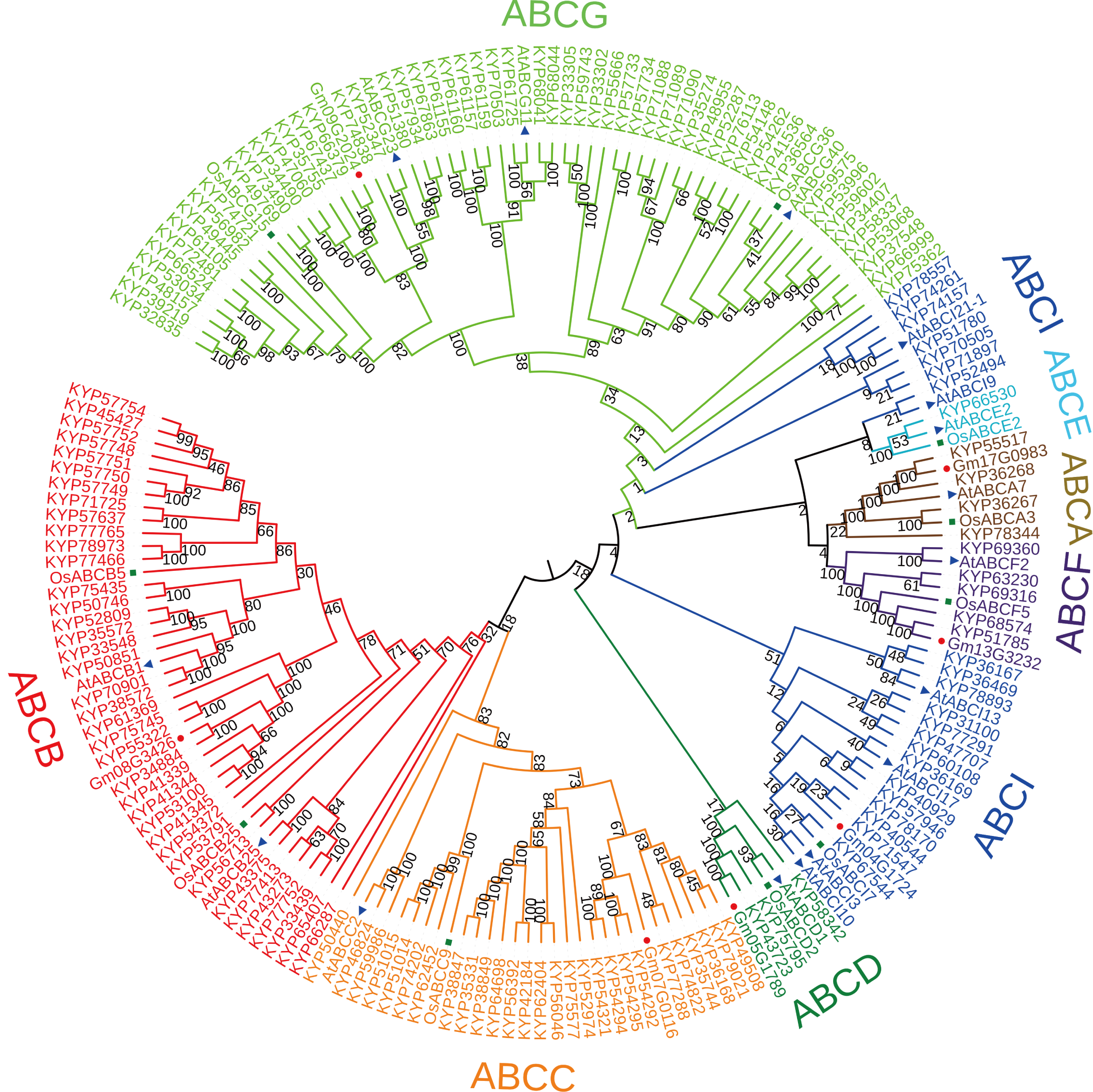

Supplement: Figure S1 — A Neighbor-Joining (NJ) tree of ABC transporters was constructed with MEGA6.0 program. The numbers beside the branches represent bootstrap values based on 1000 replications. The red circle indicates ABC transporters in soybean; the green square denotes ABC transporters in rice; the blue triangle indicates ABC transporters in Arabidopsis. All ABC transporters are divided into 8 subgroups (ABCA-ABCG, ABCI) that shown in different color at the inside of the circle. [file peerj-09-10688-s004.png]

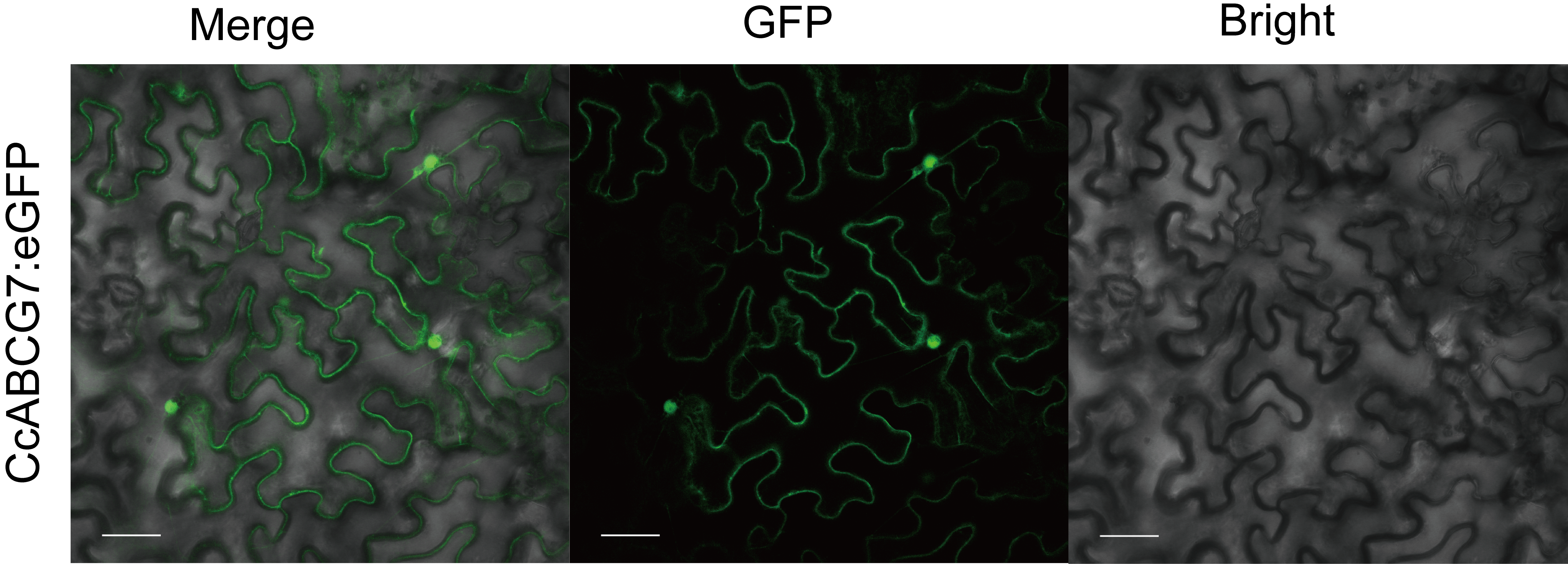

Supplement: Figure S2 — The CcABCG7 :eGFP plasmid was transformed with the into the leaves of tobacco. Fluorescence was visualized by a confocal laser scanning microscope. Bar = 50µm. [file peerj-09-10688-s005.png]

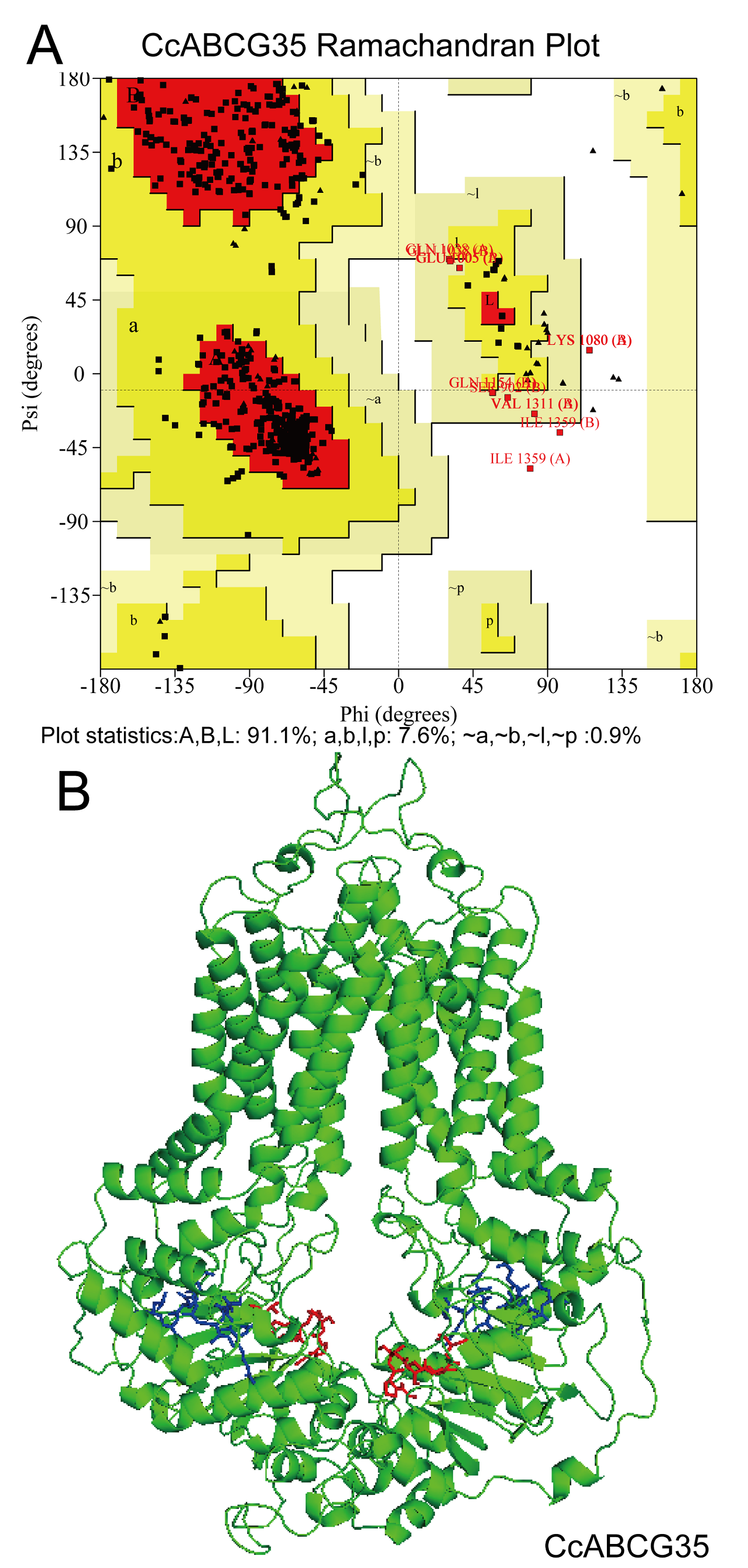

Supplement: Figure S3 — (A) In Ramachandran Plot, the A (red), B (yellow) and L (light yellow) areas represent the most favored, additional allowed and (generously allowed) areas respectively; the other (white) areas are It is a disallowed area. (B) The red and shown regions respectively represent the two motifs of the ABCG transporter NBD domain; where red represents Walker A[GPSGSGKT] and blue represents Walker B [KRVSIGQEML]. [file peerj-09-10688-s006.png]
